# Single self-cleaving mRNA vaccine expressing multiple viral structural proteins elicits robust immune responses and protects nursing piglets against PDCoV infection

**DOI:** 10.1128/jvi.00849-25

**Published:** 2025-08-06

**Authors:** Ruiming Yu, Yingjie Bai, Liping Zhang, Peng Zhou, Zhongwang Zhang, Jun Yang, Yanzhen Lu, Dongsheng Wang, Yousheng Peng, Dan Li, Jian He, Yonglu Wang, Quanwei Zhang, Ligang Yuan, Huichen Guo, Li Pan, Xinsheng Liu

**Affiliations:** 1 State Key Laboratory for Animal Disease Control and Prevention, College of Veterinary Medicine, Lanzhou Veterinary Research Institute, Chinese Academy of Agricultural Sciences, Lanzhou University 12426, Lanzhou, China; 2 Gansu Province Research Center for Basic Disciplines of Pathogen Biology, Lanzhou, China; 3 College of Veterinary Medicine, Gansu Agricultural University 739715, Lanzhou, China; 4 Hunan Institute of Animal and Veterinary Science, Changsha, China; 5 College of Life Science and Biotechnology, Gansu Agricultural University 74661, Lanzhou, China; Loyola University Chicago - Health Sciences Campus, Maywood, Illinois, USA

**Keywords:** porcine deltacoronavirus (PDCoV), spike protein, membrane protein, nucleoprotein, mRNA vaccine, immunoprotection

## Abstract

As an emerging swine enteric coronavirus, porcine deltacoronavirus (PDCoV) poses a severe threat to the global swine industry and has demonstrated potential for cross-species transmission. Therefore, the development of safe and effective vaccines is a top priority for the future prevention and control of PDCoV. In this study, we first designed and prepared an mRNA vaccine, S2P-mRNA–LNP, that expresses the full-length S2P (E855P, V856P) protein of PDCoV. Animal experiments demonstrated that S2P-mRNA–LNP induced significantly stronger humoral and cellular immune responses in mice than did an inactivated vaccine. Then, we introduced the P2A self-cleaving peptide into the S2P-mRNA–LNP design, generating SMN-mRNA–LNP, an LNP-encapsulated mRNA vaccine that allows the simultaneous expression of three major structural proteins of PDCoV (S, M, and N) from a single mRNA. The immunization of piglets demonstrated that both S2P-mRNA–LNP and SMN-mRNA–LNP induced robust humoral immune responses. Notably, SMN-mRNA–LNP conferred significantly superior active immune protection in piglets (5/5) than did S2P-mRNA-LNP (4/5). Further immunization experiments in pregnant sows showed that piglets born to SMN-mRNA–LNP-vaccinated sows acquired high levels of IgG, IgA, and neutralizing antibodies through the ingestion of colostrum, conferring complete passive protection (5/5). The protective efficacy of SMN-mRNA–LNP was markedly superior to that of the inactivated vaccine. In conclusion, these findings demonstrate that SMN-mRNA–LNP is a novel and highly efficacious candidate vaccine against PDCoV. In addition, the design strategy of single mRNA-LNP simultaneously delivering multiple viral antigens in this study provides a new idea for the development of porcine intestinal coronavirus mRNA vaccine in the future.

**IMPORTANCE:**

In this study, we designed and developed a novel mRNA vaccine, SMN-mRNA–LNP, capable of expressing the three major structural proteins (S, M, and N) of PDCoV from a single mRNA. This vaccine conferred superior active immune protection on piglets to that conferred by S2P-mRNA–LNP expressing only the S protein. Furthermore, following the immunization of pregnant sows with SMN-mRNA–LNP, their colostrum showed remarkably high IgA antibody titers reaching 1∶10^5.4^, representing a 25-fold increase over that in the inactivated vaccine group. By suckling, newborn piglets acquired significantly greater passive immunity, which ultimately conferred complete protection against PDCoV challenge.

## INTRODUCTION

Coronaviruses represent a significant group of pathogens capable of causing severe, fatal, and highly prevalent diseases in both humans and animals (1). In just over two decades in this century, humanity has already experienced three major coronavirus-induced infectious disease outbreaks, with evidence indicating that animals played a crucial role in all three (2). Research has demonstrated that many animal coronaviruses have the potential for cross-species transmission to humans, making the control of animal coronaviruses critical for public health. Porcine deltacoronavirus (PDCoV) is an emerging swine enteric pathogen that infects pigs of all ages, leading to watery diarrhea, vomiting, and intestinal pathological damage, with particularly high mortality rates in neonatal piglets (3). Since it was initially discovered and reported in 2012, PDCoV has gradually spread worldwide, posing a serious threat to the global swine industry in recent years (4). Research has shown that PDCoV can also infect chickens, turkeys, calves, and children (5–7), demonstrating its potential for cross-species transmission and the significant risk it poses to public health security. Vaccination remains the most effective strategy for preventing and controlling this disease. However, no commercial vaccine against PDCoV is currently available. Therefore, the development of safe and highly effective vaccines has become an urgent requirement for PDCoV prevention and control.

The development strategies for coronavirus vaccines primarily include traditional inactivated vaccines, live-attenuated vaccines, and novel genetically engineered vaccines. The latter includes subunit vaccines, nucleic acid vaccines, and viral vector vaccines (1). In recent years, the unique advantages of the mRNA vaccine technology have demonstrated revolutionary potential in the prevention and control of infectious diseases: short development cycles, ease of industrialization, simplified production processes, rapid adaptability to emerging variants, and the capacity to induce both humoral and cellular immune responses (8). To date, mRNA vaccines have been applied in research into the prevention and control of diseases, such as COVID-19, influenza, and dengue fever, and have demonstrated particularly significant efficacy in combating the COVID-19 pandemic.

PDCoV is classified within the genus *Deltacoronavirus* in the family *Coronaviridae* and the order *Nidovirales* and is a single-stranded positive-sense RNA virus. The mature virion contains four structural proteins: the spike (S) glycoprotein, membrane (M) protein, nucleoprotein (N), and envelope (E) protein (9). The S protein, a type I transmembrane glycoprotein that forms spike-like projections on the viral surface, primarily mediates host–receptor recognition, binding, and membrane fusion for viral entry (10). It exists in prefusion and postfusion conformations, and the prefusion state is the primary conformation that induces neutralizing antibodies. The introduction of two consecutive proline mutations at the turn between the central helix of the S2 subunit and heptad repeat 1 (HR1) stabilizes the highly immunogenic prefusion state, thereby inducing stronger immune responses. This design strategy has been implemented in various coronavirus vaccines, including the mRNA vaccines of Moderna/BioNTech, Johnson & Johnson’s adenoviral vector vaccine, and Novavax’s recombinant protein vaccine (11–14). The M protein, the most abundant transmembrane protein in the coronavirus envelope, plays essential roles in viral assembly and budding and displays high conservation across different coronaviruses (15). During viral entry, it critically mediates interactions between the viral envelope and host-cell membranes (16). Studies have shown that the M protein contains both T-cell epitopes and neutralizing antibody epitopes and can induce robust T-cell responses, making it a potential target for coronavirus vaccine development (17, 18). The N protein, the most abundant and multifunctional coronavirus protein, plays vital roles in viral replication. Although vaccine development has primarily focused on the S protein, emerging evidence suggests that the N protein can also be used as a major target for antibody responses and T-cell epitopes. N-protein-based vaccines induce potent humoral and cellular immune responses, protecting against coronavirus infections (1, 19, 20). Therefore, the N protein represents another promising candidate vaccine antigen. Current coronavirus mRNA vaccine research predominantly focuses on the single S protein antigen. However, during natural infections, both the M and N proteins participate in critical immune responses. These proteins contain abundant, conserved T-cell epitopes, which may enhance cross-protective immunity. Therefore, the designs of multi-antigen mRNA vaccines incorporating these components could overcome the limitations of single-target approaches, potentially improving the breadth and durability of immune protection through their synergistic effects.

In this study, we propose for the first time a PDCoV mRNA vaccine strategy based on the combination of three structural proteins of PDCoV—S, M, and N—linked by the self-cleaving peptide P2A, together with proline mutations in the S protein. In this way, a single mRNA can express multiple antigens of PDCoV. We hoped to take advantage of the ability of the S protein to induce neutralizing antibodies, the immunomodulatory functions of the M protein, and the cellular immune activation properties of the N protein to build a hierarchical defense system. We comprehensively evaluated the vaccines’ immunogenicity, antibody responses, and protective efficacy against viral challenge in murine models, nursing piglets, and pregnant sows to establish a novel strategy for developing broad-spectrum, high-efficacy PDCoV vaccines and to provide empirical evidence to advance research into multi-antigen mRNA vaccines against coronaviruses.

## RESULTS

### Single mRNA expresses three structural proteins (S, M, and N) of PDCoV

**Fig 1:**
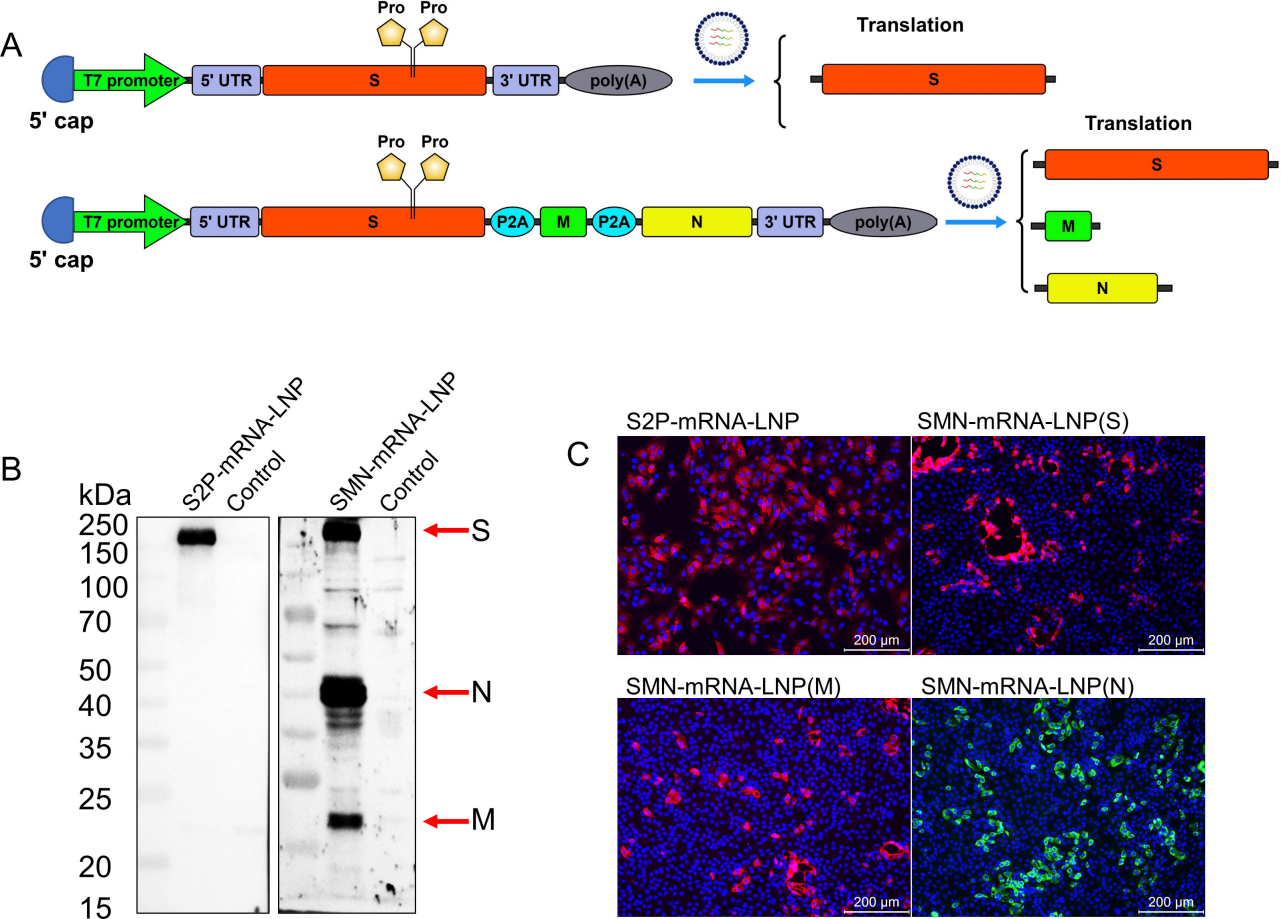
PDCoV mRNA vaccine design and confirmation of expression. (**A**) Schematic diagram of the design and expression of the mRNA sequence. The noncoding regions of both mRNA sequences contain the 5′-cap, 5′-untranslated region (UTR), 3′-UTR, and 3′-polyA tail elements. The S2P-mRNA–LNP coding sequence (CDS) contains only the S gene with a double proline mutation, whereas the SMN-mRNA-LNP CDS region contains three genes encoding antigens S, M, and N and is connected in series with the P2A self-cleaving peptide. (**B**) *In vitro* expression of S2P-mRNA–LNP and SMN-mRNA–LNP was confirmed with western blotting. 293T or LLC-PK cells were treated with S2P-mRNA–LNP or SMN-mRNA–LNP. After 24 h, the cells were lysed and analyzed with western blotting, probed with rabbit anti-S, -M, or -N protein polyclonal antibody as the primary antibody and HRP-conjugated goat anti-rabbit IgG as the secondary antibody. (**C**) *In vitro* expression of S2P-mRNA–LNP and SMN-mRNA–LNP was confirmed with an immunofluorescent assay (IFA). LLC-PK cells were treated with S2P-mRNA–LNP or SMN-mRNA–LNP. After 24 h, the cells were fixed with 4% paraformaldehyde, and IFA was performed with rabbit anti-S or -M polyclonal antibody or mouse anti-N monoclonal antibody as the primary antibody, and Alexa-Fluor-594-conjugated goat anti-rabbit IgG or Alexa-Fluor-488-conjugated goat anti-mouse IgG as the secondary antibody.

The P2A self-cleaving peptide is a short peptide with a self-cleaving function, which allows the translation of multiple peptide chains from a single mRNA, and ultimately the expression of multiple proteins. We inserted the PDCoV S, M, and N coding sequences (CDSs) in the same open reading frame (ORF) and introduced them into the P2A self-cleaving peptide. To stabilize the prefusion conformation of the S protein to improve its immunogenicity, we introduced two consecutive proline mutations (E855P, V856P) into the turn between the central helix of the S2 subunit and HR1, to form the S2P protein. This ultimately allowed three structural proteins, S, M, and N, to be expressed in a single mRNA. Two PDCoV mRNA vaccines were designed and prepared: S2P-mRNA–LNP and SMN-mRNA–LNP (Fig. 1A). 293T and LLC-PK cells were transfected with the two prepared lipid nanoparticles (LNPs), and western blotting and immunofluorescence assay (IFA) analyses confirmed that S2P-mRNA–LNP correctly expressed the S protein and SMN-mRNA–LNP correctly expressed the three structural proteins, S, M, and N (Fig. 1B and C).

### S2P-mRNA–LNP induces a stronger immune response than the inactivated PDCoV vaccine in mice

**Fig 2:**
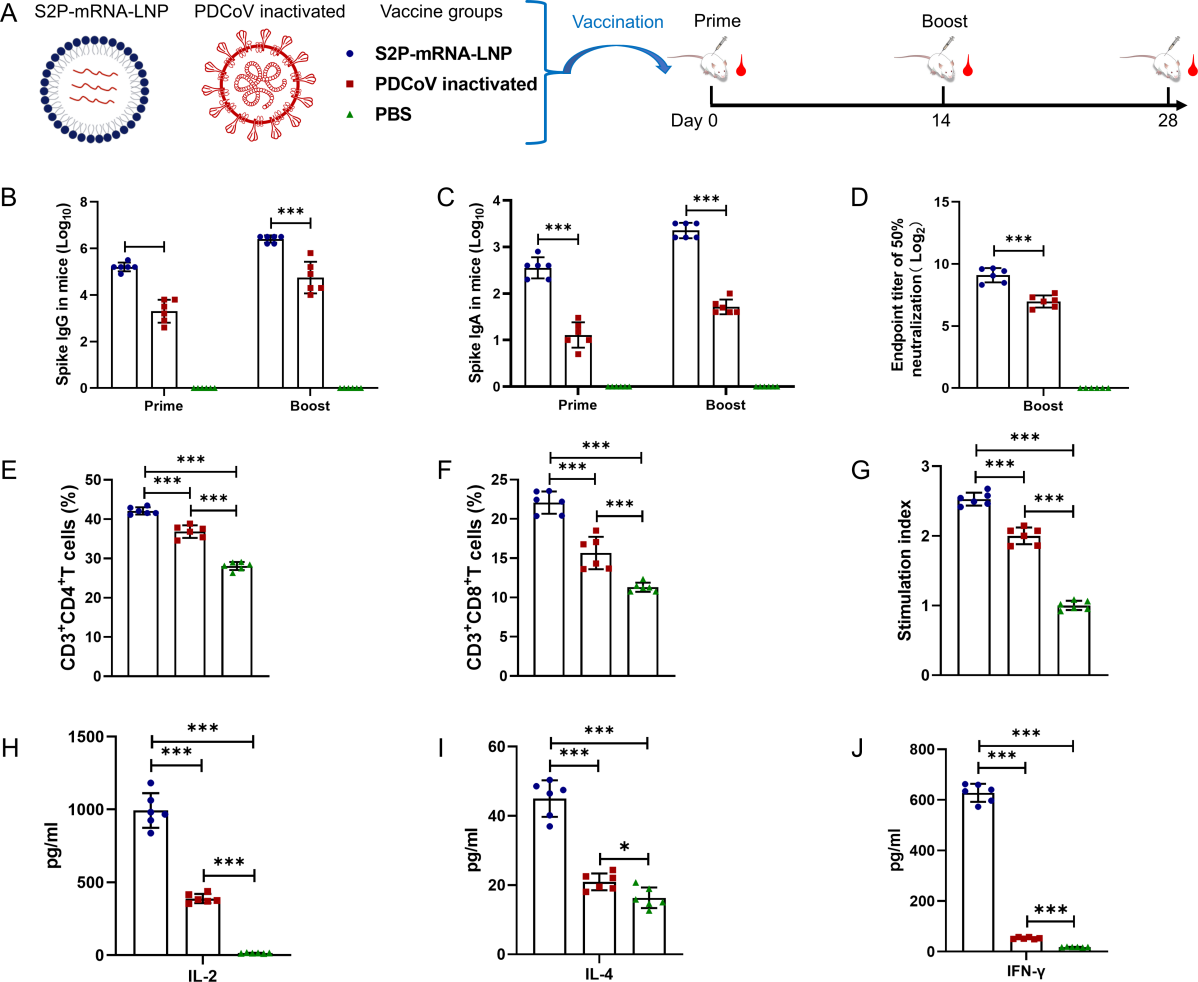
Immunogenicity of S2P-mRNA–LNP in mice. (**A**) Immune grouping and immune procedure of mice. Mice were randomly divided into three groups and immunized with S2P-mRNA–LNP, inactivated PDCoV vaccine, or PBS. The mice were immunized once on days 0 and 21, and blood was collected from the eyeball veins on days 14 and 28. (**B, C**) Levels of S-protein-specific IgA and IgG antibodies in sera of mice 14 days after primary and booster immunizations. (**D**) PDCoV (strain CH/XJYN/2016) neutralization assay showed the half-maximal neutralizing antibody titer (NT50). The neutralizing antibody titer in the sera of mice was determined with the fixed virus–diluted serum method. Neutralizing antibody titers were calculated as the reciprocal of the highest serum dilution that inhibited 50% CPEs. (E–J) Characteristics of the cellular immune response in mice 28 days after immunization. Percentages of CD3^+^CD4^+^ (**E**) and CD3^+^CD8^+^ (**F**) T cells in mouse splenic lymphocytes were determined with flow cytometry. The lymphocytes were stimulated with S protein for 72 h, and the proliferation rate was measured with a CCK-8 kit (**G**). S protein was used to stimulate lymphocytes to secrete cytokines. The supernatants of the cell cultures were collected, and IL-2, IL-4, and IFN-γ were detected with ELISAs (H to J). Data are the means ± SD of six mice per group. Asterisks in the figures indicate significant differences: *P < 0.05, statistically significant difference; ***P < 0.01, very highly significant difference.

To evaluate the immune responses induced by the mRNA–LNP vaccines *in vivo*, we first assessed the immunogenicity of S2P-mRNA–LNP in mice and compared it with that of the inactivated PDCoV vaccine (Fig. 2A). Both the S2P-mRNA–LNP and inactivated vaccine groups generated PDCoV S-specific IgG and IgA antibodies after the primary immunization—whereas the PBS control did not—and the responses were boosted following the second dose. The S2P-mRNA–LNP group showed significantly higher antibody titers than the inactivated vaccine group at all time points, particularly for IgA (Fig. 2B and C). Neutralization assays revealed substantially higher neutralizing antibody titers in the S2P-mRNA–LNP group than in the inactivated vaccine group after both rounds of immunization (Fig. 2D).

To assess the level of cellular immunity induced by S2P-mRNA–LNP and the inactivated PDCoV vaccines in mice, mouse spleen lymphocytes were isolated 28 days after priming. The T-lymphocyte subsets were identified and counted with flow cytometry. Eukaryotic-expressed PDCoV S protein was used to stimulate lymphocyte proliferation *in vitro*, and ELISAs were used to determine the levels of IFN-γ, IL-2, and IL-4 in the lymphocyte culture supernatants. The percentages of CD3^+^CD4^+^ and CD3^+^CD8^+^ T cells in the S2P-mRNA–LNP and inactivated PDCoV vaccine groups were significantly higher than those in the PBS control group, and the percentages of CD3^+^CD4^+^ and CD3^+^CD8^+^ T cells in the S2P-mRNA–LNP group were significantly higher than those in the inactivated PDCoV vaccine group (Fig. 2E and F). The lymphocyte stimulation indices of the S2P-mRNA–LNP and inactivated PDCoV vaccine groups were significantly higher than that of the PBS control group, and the stimulation index of the S2P-mRNA–LNP group was significantly higher than that of the inactivated PDCoV vaccine group (Fig. 2G). The levels of cytokines IFN-γ, IL-2, and IL-4 were significantly higher in the S2P-mRNA–LNP group than in the inactivated PDCoV vaccine group (Fig. 2H through J). These data suggest that S2P-mRNA–LNP induces stronger humoral and cellular immune responses than the inactivated PDCoV vaccine.

### SMN-mRNA–LNP induces better active immunoprotection in piglets than S2P-mRNA–LNP

**Fig 3:**
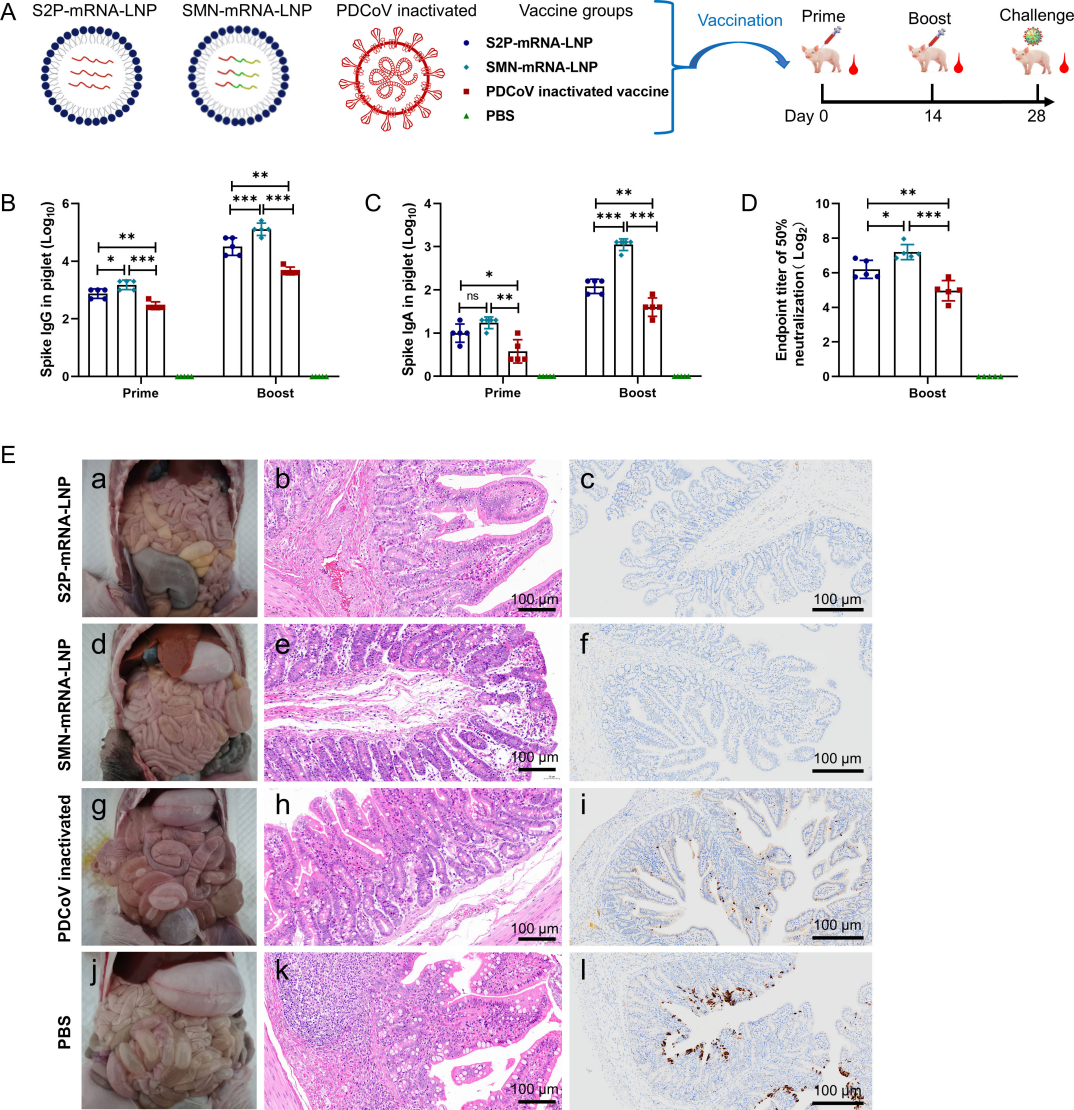
Active protective immune responses to S2P-mRNA–LNP and SMN-mRNA–LNP in piglets. (**A**) Immune grouping and immune procedure of piglets. Piglets were randomly divided into four groups and immunized with S2P-mRNA–LNP, SMN-mRNA–LNP, inactivated PDCoV vaccine, or PBS. The piglets were immunized once on days 0 and 14, and blood was collected from eyeball veins on days 14 and 28. (**B, C**) Levels of S-protein-specific IgA and IgG antibodies in the sera of the piglets 14 days after primary and booster immunizations. (**D**) Neutralizing antibody titers in the sera of piglets 28 days after active immunization. (**E**) Clinical diarrhea, gross, intestinal histopathology, and immunohistochemical examination of euthanized piglets 7 days after challenge. Representative intestinal sections are shown after postmortem examination, hematoxylin–eosin (H&E) staining, and immunohistochemistry. PDCoV antigen signal is brown. (a–c) Examination of piglets in S2P-mRNA–LNP group. (d–f) Examination of piglets in SMN-mRNA–LNP group. (g–i) Examination of piglets in the inactivated PDCoV vaccine group. (j–l) Examination of piglets in PBS group. Data are the means ± SD of five piglets per group. Asterisks in the figures indicate significant differences: *P < 0.05, significant difference; **P < 0.01, highly significant difference; ***P < 0.001, very highly significant difference; ns, not significant.

We designed an mRNA vaccine in which a single mRNA expressed three antigens of PDCoV, designated SMN-mRNA–LNP, and compared its active immunoprotective effects in piglets with those of S2P-mRNA–LNP and an inactivated PDCoV vaccine (Fig. 3A). High levels of PDCoV-S-protein-specific IgG and IgA antibodies were induced in the SMN-mRNA–LNP, S2P-mRNA–LNP, and inactivated PDCoV vaccine groups after immunization, but not in the PBS control group. After the booster immunization, both the IgG and IgA antibody titers induced were significantly higher in the SMN-mRNA–LNP group than in the S2P-mRNA–LNP or inactivated PDCoV vaccine group (Fig. 3B and C). An *in vitro* neutralization assay showed that SMN-mRNA–LNP induced higher neutralizing antibody titers than either the S2P-mRNA–LNP or inactivated PDCoV vaccine (Fig. 3D).

To assess the active immunoprotection conferred on piglets by the mRNA vaccines in this study, a protection test was performed on day 28 after the two-dose immunization of piglets. The clinical signs and fecal detoxification of the piglets after oral vaccination with 8 mL × 10^4.0^ TCID_50_/mL PDCoV strain CH/XJYN/2016 are shown in Table 1. The piglets in the S2P-mRNA–LNP group, which were vaccinated at 4 days post-infection (dpi; CT = 26.81 ± 3.87; FC = 0.2 ± 0.45) and 5 dpi (CT = 28.40 ± 2.79; FC = 0.2 ± 0.45), showed mild diarrhea and recovered at 6 dpi, with the disappearance of diarrhea symptoms. All piglets in the SMN-mRNA–LNP group were in good condition on 0–7 dpi, with a normal appetite and no diarrhea symptoms (FC = 0), and slight detoxification was detected in anal swabs on 3–5 dpi. One piglet in the inactivated PDCoV vaccine group showed slight diarrhea at 3 dpi (CT = 21.01 ± 6.85; FC = 0.20 ± 0.45), and two pigs had diarrhea symptoms at 4 dpi (CT = 23.64 ± 3.92; FC = 0.60 ± 0.89) and there were three cases of diarrhea symptoms at 5 dpi (CT = 23.10 ± 4.24; FC = 1.00 ± 1.00). In the PBS group, one piglet showed clinical symptoms at 3 dpi, and all piglets in the PBS group showed diarrhea symptoms at 4 dpi (CT = 21.13 ± 2.63; FC = 1.40 ± 0.55), which continued until 6 dpi. Clinical observation and monitoring for 7 days after challenge revealed that the S2P-mRNA–LNP-immunized piglets had a protection rate of 4/5, the SMN-mRNA–LNP-immunized piglets had a protection rate of 5/5, the inactivated-PDCoV-immunized piglets had a protection rate of 2/5, and all the piglets in the PBS group developed disease (5/5).

**TABLE 1:** Summary of the clinical scores and fecal viral shedding of piglets challenged with 8 mL ×10^4.0^ TCID_50_/mL PDCoV CH/XJYN/2016 after active immunization

dpi^*a*^	S2P-mRNA-LNP (*n* = 5)			SMN-mRNA-LNP (*n* = 5)			PDCoV inactivated (*n* = 5)			PBS (*n* = 5)		
	CT^*b*^	FC^*c*^	NP^*d*^	CT	FC	NP	CT	FC	NP	CT	FC	NP
0	33.86 ± 1.35	0	0/5	33.15 ± 1.97	0	0/5	33.06 ± 0.94	0	0/5	33.37 ± 0.53	0	0/5
1	31.19 ± 1.63	0	0/5	30.69 ± 0.55			27.73 ± 5.37	0	0/5	28.63 ± 3.15	0	0/5
2	28.60 ± 2.54	0	0/5	30.15 ± 3.49			24.40 ± 6.76	0	0/5	25.53 ± 3.30	0.40 ± 0.89	1/5
3	27.41 ± 4.06	0	0/5	27.24 ± 4.69			21.01 ± 6.85	0.20 ± 0.45	1/5	22.73 ± 2.18	0.80 ± 0.84	3/5
4	26.81 ± 3.87	0.20 ± 0.45	1/5	29.56 ± 2.73			23.64 ± 3.92	0.60 ± 0.89	2/5	21.13 ± 2.63	1.40 ± 0.55	5/5
5	28.40 ± 2.79	0.20 ± 0.45	1/5	29.91 ± 2.63			23.10 ± 4.24	1.00 ± 1.00	3/5	23.25 ± 2.07	1.20 ± 0.45	5/5
6	29.05 ± 2.33	0	0/5	29.74 ± 1.58			26.84 ± 4.54	0.4 ± 0.89	1/5	24.58 ± 2.61	0.40 ± 0.55	2/5
7	29.42 ± 2.02	0	0/5	30.26 ± 1.68			28.74 ± 2.50	0	0/5	27.61 ± 1.96	0	0/5

^*a*^ Days post-inoculation.

^*b*^ The CT mean value for each group; a cutoff point was set at 30; CT values greater than 30 were considered negative or below the detection limit of RT-PCR.

^*c*^ Clinical score for fecal consistency, as follows: 0 = normal; 1 = pasty; 2 = semiliquid; and 3 = liquid.

^*d*^ Number of PDCoV-positive piglets.

The piglets were dissected and sampled 7 days after challenge. The piglets in the SMN-mRNA–LNP and S2P-mRNA–LNP groups had normal intestinal tissues with no obvious lesions; the piglets in the inactivated PDCoV vaccine group had slightly thinned intestines and a small amount of yellowish fluid in the intestinal lumen; and the piglets in the PBS group had thinned, transparent intestines and a large amount of yellowish fluid in the intestinal lumen (Fig. 3E). Ileal tissues were selected for histopathology and immunohistochemistry. Histopathology revealed no obvious intestinal pathological damage in the piglets of the SMN-mRNA–LNP and S2P-mRNA–LNP groups. The piglets in the inactivated PDCoV vaccine group showed edema of the intestinal villous epithelial cells, a low number of cup cells, increased edema in the lamina propria, and increased edema in the submucosal layer. The piglets in the PBS group had a greater number of the intestinal villous epithelial cells that were edematous, the cytosol was swollen, the number of cup cells was small, the connective tissue and intestinal glands were loosely arranged, occasional granulocyte infiltration was detected, and the muscular layer of the mucous membrane separated the intestinal crypts from the submucosal layer (Fig. 3E). An immunohistochemical analysis showed that the intestines of the piglets in the SMN-mRNA–LNP and S2P-mRNA–LNP groups were almost free of PDCoV antigens, whereas large amounts of PDCoV antigens were detected in the intestinal epithelial cells of the piglets in the inactivated PDCoV vaccine group and PBS group (Fig. 3E).

### SMN-mRNA–LNP induces strong immune responses in sows

**Fig 4:**
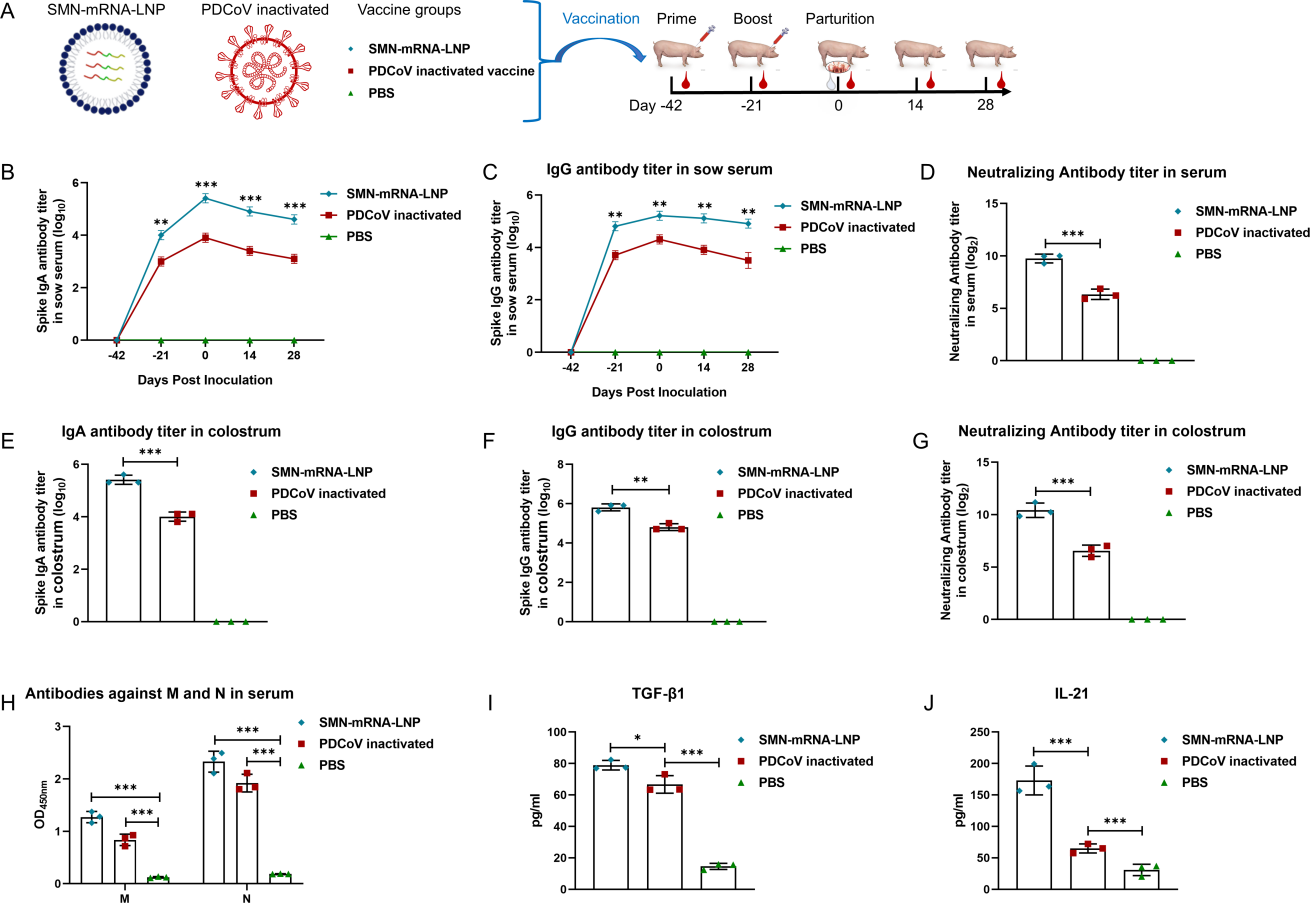
Immunogenicity of SMN-mRNA–LNP in pregnant sows. (**A**) Immune grouping and immune procedure of pregnant sow. Pregnant sows were randomly divided into three groups and immunized with SMN-mRNA–LNP, inactivated PDCoV vaccine, or PBS. The pregnant sows were immunized on days 42 and 21 before delivery. Blood was collected on days 42 and 21 before delivery, on the day of delivery, and 14 and 28 days after delivery. Colostrum was collected 2 h after delivery. (**B, C**) Dynamic changes in IgG and IgA antibody levels in pregnant sow sera before and after immunization. (**D**) Neutralizing antibody titers in the sera of pregnant sows after two immunizations. (E–G) IgA, IgG, and neutralizing antibody titers in colostrum of sows. (**H**) PDCoV M and N protein antibody titers in the sera of pregnant sows after two immunizations. (**I, J**) S protein stimulated lymphocytes to secrete cytokines. Supernatants of cell cultures were collected, and TGF-β and IL-21 were detected with ELISAs. Data are the means ± SD of three sows per group. Asterisks in the figures indicate significant differences: *P < 0.05, significant difference; **P < 0.01, highly significant difference; ***P < 0.001, very highly significant difference; ns, not significant.

The results of the active immunoprotection study in piglets indicated that SMN-mRNA–LNP is superior to S2P-mRNA–LNP in that context. Therefore, we further evaluated the immunogenicity of SMN-mRNA–LNP in gestating sows and compared it with that of the inactivated PDCoV vaccine. The results are shown in Fig. 4. After two immunizations, PDCoV-S protein-specific IgA and IgG antibodies were detected in the sera and colostrum of sows in the SMN-mRNA–LNP and inactivated PDCoV vaccine groups, and the IgA and IgG antibodies in the sera remained high on days 14 and 28 after farrowing. The IgA and IgG antibody titers in the sera and colostrum of the sows in the SMN-mRNA–LNP group were significantly higher than those in the inactivated PDCoV vaccine group (Fig. 4B, C, E and F). An *in vitro* neutralization assay showed that the neutralizing antibody titers in the sera and colostrum of sows in the SMN-mRNA–LNP group were significantly higher than those in the sows in the inactivated PDCoV vaccine group (Fig. 4D and G). Specific antibodies directed against the M and N proteins were also detected in the sera of sows in the SMN-mRNA–LNP and inactivated PDCoV vaccine groups, but were significantly higher in the SMN-mRNA–LNP group than in the inactivated PDCoV vaccine group (Fig. 4H), indicating that our SMN-mRNA–LNP vaccine effectively expressed the three antigens of PDCoV S, M, and N in the sows and induced high levels of specific antibodies. We examined two key cytokines (TGF-β1 and IL-21) that regulate IgA and found that SMN-mRNA–LNP induced the production of higher levels of TGF-β1 and IL-21 than the inactivated PDCoV vaccine (Fig. 4I and J).

### SMN-mRNA–LNP confers complete passive immunoprotection on piglets

**Fig 5:**
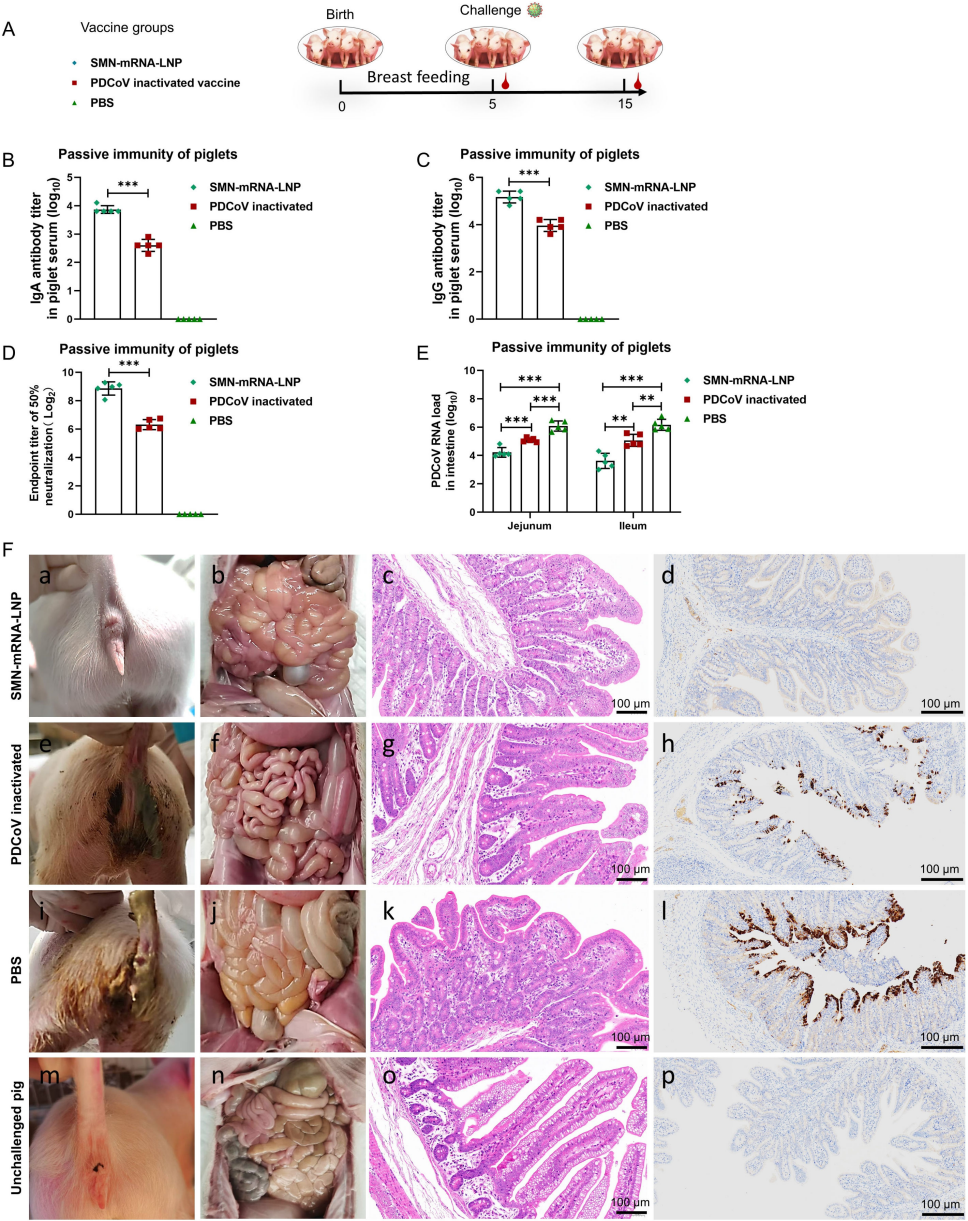
Protective effect of passive immunity induced in piglets by SMN-mRNA–LNP. (**A**) Schematic diagram of the challenge of passively immunized piglets. After being breastfed for 5 days, all newborn piglets were challenged with PDCoV strain CH/XJYN/2016 and euthanized at 10 days post-challenge. (B–D) Levels of antibodies, derived from breast milk, in newborn piglets. The blood of newborn piglets was collected after they had consumed breast milk for 5 days, and the serum was separated. Levels of IgA, IgG, and neutralizing antibodies passively acquired by the piglets were determined. (**E**) Real-time fluorescence quantitative PCR (RT-qPCR) was used to analyze the viral RNA copy in the intestinal tissue of piglets after challenge. (**F**) Clinical diarrhea, gross, intestinal histopathology, and immunohistochemical examination of euthanized piglets 10 days after challenge. Representative intestinal sections are shown after post-mortem examination, H&E staining, and immunohistochemical assay. PDCoV antigen signal is brown. (a–d) Examination of piglets delivered by sows in the SMN-mRNA-LNP group. (e–h) Examination of piglets delivered by sows in the inactivated PDCoV vaccine group. (i–l) Examination of piglets delivered by sows in the PBS group. (m–p) Examination of healthy unchallenged pigs. Data are means ± SD of five newborn piglets per group. Asterisks in the figures indicate significant differences: *P < 0.05, significant difference; ***P < 0.001, very highly significant difference.

Nursing piglets are protected by passive immunization when they ingest protective antibodies in the sow’s milk. Sows immunized with SMN-mRNA–LNP or inactivated PDCoV vaccines were evaluated for their capacity to provide passive immunoprotection by orally vaccinating them with 1 mL of 10^4.0^ TCID_50_/mL PDCoV strain CH/XJYN/2016 after delivery and allowing their piglets to suckle their breast milk naturally for 5 days. The piglets in the SMN-mRNA–LNP group passively acquired significantly higher titers of IgA, IgG, and neutralizing antibodies than those in the inactivated PDCoV vaccine group (Fig. 5B through D). As shown in Table 2 and Table S1, after challenge, all piglets in the SMN-mRNA–LNP group were in good condition at 0-10 dpi, had a normal appetite, and no diarrhea symptoms (FC score = 0). Piglets in the inactivated PDCoV vaccine group began to show symptoms of mild diarrhea, vomiting, and loss of appetite at 2 dpi. PDCoV RNA shedding was detected in the feces (CT = 28.36 ± 4.05; FC = 0.80 ± 1.10) and continued to 7 dpi. At 8 dpi, the sick piglets in the inactivated PDCoV vaccine group recovered with no clinical symptoms, such as diarrhea, but PDCoV RNA shedding was still detected in their feces (CT = 25.12 ± 0.46; FC = 0). Three piglets in the PBS group showed symptoms of diarrhea, vomiting, and loss of appetite at 2 dpi. PDCoV RNA shedding was detectable in their feces (CT = 24.87 ± 5.24, FC = 1.80 ± 1.64), and diarrhea symptoms were observed in all piglets from 3 dpi (CT = 22.80 ± 1.98; FC = 1.80 ± 0.45) until 9 dpi. The piglets in the PBS group recovered with no clinical symptoms, such as diarrhea, but PDCoV RNA shedding was still detectable in the feces (CT = 25.65 ± 3.88; FC = 0). Clinical observation and monitoring for 10 days after challenge revealed that the passive protection rate in the piglets of the SMN-mRNA–LNP group was 5/5, and that in the inactivated PDCoV vaccine group was 2/5, whereas all the piglets in the PBS group developed disease (5/5). Pathological histological examination and immunohistochemistry also detected no pathological damage in the intestines of the piglets in the SMN-mRNA–LNP group (Fig. 5F), and no PDCoV antigen was detected in them. In the inactivated PDCoV vaccine group, a slight thinning of the intestinal wall was seen, with a small amount of fluid in the intestinal lumen and a small amount of PDCoV antigen in intestinal epithelium. In the PBS group, a thinning of the intestinal wall was seen, with large amounts of fluid in the intestinal lumen and large amounts of PDCoV antigens in the intestinal epithelial cells.

**TABLE 2:** Summary of the clinical scores and fecal viral shedding of piglets challenged with 1 mL ×10^4.0^ TCID_50_/mL PDCoV CH/XJYN/2016 after passive immunization

dpi^*a*^	SMN-mRNA-LNP (*n* = 5)			PDCoV inactivated (*n* = 5)			PBS (*n* = 5)		
	CT^*b*^	FC^*c*^	NP^*d*^	CT	FC	NP	CT	FC	NP
0	33.82 ± 1.64	0	0/5	33.12 ± 1.28	0	0/5	34.10 ± 1.13	0	0/5
1	32.47 ± 0.68			32.15 ± 0.80	0	0/5	29.04 ± 1.61	0	0/5
2	32.69 ± 1.34			28.36 ± 4.05	0.80 ± 1.10	2/5	24.87 ± 5.24	1.80 ± 1.64	3/5
3	31.07 ± 0.59			22.10 ± 2.45	0.80 ± 1.10	2/5	22.80 ± 1.98	1.80 ± 0.45	5/5
4	30.68 ± 1.69			21.67 ± 0.94	1.60 ± 1.52	3/5	21.93 ± 1.75	1.20 ± 0.45	5/5
5	28.31 ± 1.07			22.10 ± 5.23	1.60 ± 1.52	3/5	23.51 ± 2.53	2.00 ± 0.71	5/5
6	29.28 ± 1.99			20.92 ± 3.08	1.00 ± 1.41	2/5	22.00 ± 2.12	0.80 ± 0.84	3/5
7	27.60 ± 0.83			22.50 ± 0.24	0.40 ± 0.89	1/5	24.30 ± 3.43	0.80 ± 0.84	3/5
8	29.24 ± 1.57			25.12 ± 0.46	0	0/5	24.04 ± 3.52	0.60 ± 0.89	2/5
9	30.66 ± 1.95			28.52 ± 3.18	0	0/5	25.65 ± 3.88	0	0/5
10	31.67 ± 0.70			28.21 ± 1.64	0	0/5	25.81 ± 3.22	0	0/5

^*a*^ Days post-inoculation.

^*b*^ The CT mean value for each group; a cutoff point was set at 30; CT values greater than 30 were considered negative or below the detection limit of RT-PCR.

^*c*^ Clinical score for fecal consistency, as follows: 0 = normal; 1 = pasty; 2 = semiliquid; and 3 = liquid.

^*d*^ Number of PDCoV-positive piglets.

## DISCUSSION

Porcine enteric viral diseases cause significant economic losses in the swine industry, and vaccination has been one of the most effective means of controlling and preventing infectious diseases. Traditional inactivated and live-attenuated vaccines play an important role in the prevention and control of infectious diseases. mRNA vaccines are an emerging type of nucleic acid vaccine, and immunization with this type of vaccine induces both humoral and cellular immunity (21). The successful marketing of the COVID-19 mRNA vaccine signifies the gradual maturation of this type of vaccine in terms of the technological systems required. mRNA vaccines show great potential utility in the prevention of infectious diseases, especially emerging infectious diseases, due to their advantages of rapid deployment and a short development cycle (22). In this study, as an innovation, we incorporated the PDCoV M and N antigens into the vaccine design and developed the mRNA vaccine SMN-mRNA–LNP, in which a single mRNA expresses three antigens of PDCoV—S, M, and N. We then evaluated its effectiveness in the active and passive immunoprotection of piglets. Our results show that SMN-mRNA–LNP provides complete active and passive immunoprotection to piglets and is a very promising vaccine for PDCoV. This mRNA vaccine design can also be applied to the development of other coronavirus vaccines.

The S protein is a key structural protein of coronaviruses, which mediates the recognition of host-cell receptors and viral entry. It displays strong immunogenicity and induces high levels of protective antibodies in the host, making it the preferred target antigen for vaccine development (10, 23). In this study, we first designed an mRNA vaccine—S2P-mRNA–LNP—based on the full-length PDCoV S protein, with a double proline mutation introduced to stabilize its prefusion conformation. After their immunization with S2P-mRNA–LNP, mice produced high levels of S-protein-specific IgG, IgA, and neutralizing antibodies, indicating that the mutated S protein was correctly expressed and induced a strong humoral immune response. Immediately thereafter, we examined the cellular immunity level in the mice and showed that S2P-mRNA–LNP induced stronger cellular immunity than the inactivated PDCoV vaccine. Li et al. (24) have also shown that an mRNA vaccine based on the PDCoV S protein induced high levels of IgG and neutralizing antibodies in mice, and that it also conferred better protection than the inactivated vaccine on passively immunized nursing piglets. The S mRNA vaccine also conferred better protection than an inactivated vaccine on passively immunized nursing piglets. Similarly, the immunization of mice with the COVID-19 mRNA vaccine BNT162b2 stimulated the production of potent antibodies and antigen-specific T-cell responses and significantly enhanced the intrinsic immune response after a second immunization. mRNA-vaccine-conferred protection against SARS-CoV-2 infection is mediated primarily by humoral immunity, whereas cellular immunity effectively clears the infection (25).

In a previous study (26), we found that the immunization of mice or piglets with a mixture of the three PDCoV antigens, S, M, and N, induced stronger humoral and cellular immune responses than immunization with S alone and was effective in protecting piglets against PDCoV, with an immunoprotection rate of 80% (4/5). In a study of transmissible gastroenteritis virus (TGEV), Sestak et al. (27) also showed that immunization with a mixture of S, M, and N effectively stimulated sows to produce a T-cell immune response that protected piglets from TGEV. Therefore, we also incorporated both PDCoV M and N antigens into the mRNA vaccine design, generating the PDCoV mRNA vaccine SMN-mRNA–LNP, in which a single mRNA expresses all three antigens simultaneously. We compared and evaluated the active immunoprotective effects of SMN-mRNA–LNP, S2P-mRNA–LNP, and inactivated PDCoV vaccine in piglets. Both SMN-mRNA–LNP and S2P-mRNA–LNP induced the production of high levels of PDCoV S-protein-specific IgG and IgA antibodies, indicating that both SMN-mRNA–LNP and S2P-mRNA–LNP correctly expressed the S antigen in the piglets. However, the SMN-mRNA–LNP-induced IgG, IgA, and neutralizing antibody titers were significantly higher than those induced by S2P-mRNA–LNP. The results of a challenge experiment showed that SMN-mRNA–LNP provided 100% active immunoprotection to piglets, whereas S2P-mRNA–LNP conferred only partial protection. Pathological tissue examination and immunohistochemistry also showed that the piglets in the SMN-mRNA–LNP group had less intestinal PDCoV antigen and less pathological damage than those in the S2P-mRNA–LNP group. Previous studies of COVID-19 have also shown that COVID-19 vaccines based on the SARS-CoV-2 S and N proteins provided acute protection in the lungs and brain (20). Therefore, coronavirus vaccines based on multiple structural proteins are significantly superior to coronavirus vaccines based on only a single structural protein in terms of the level of immunoprotective afforded.

The main danger of porcine enteroviruses is the massive mortality in newborn piglets, so the protection of piglets from enteroviruses is an urgent problem in veterinary clinics (28). Most infectious diseases of piglets occur within 1 week of birth, when the piglets’ immune systems are not yet mature, and the vaccination of piglets at this time is ineffective. Therefore, the focus of prevention and control must be on the immunization of gestating sows so that the piglets can be protected by passive immunity acquired through the sows’ colostrum. After sows were immunized with SMN-mRNA–LNP, higher S-antigen-specific IgG, IgA, and neutralizing antibody titers were detected in their colostrum and sera than in those of sows vaccinated with an inactivated vaccine. This was especially true of their colostrum, and the IgA antibody titers in the colostrum from sows in the SMN-mRNA–LNP group were as high as 1∶10^5.4^, 25 times higher than those in the inactivated vaccine group. Studies have shown that TGF-β1 and IL-21 are key cytokines regulating porcine IgA production (29–31), so we examined the cytokines TGF-β1 and IL-21 produced by the sows’ peripheral blood lymphocytes. Higher levels of TGF-β1 and IL-21 were detected in the SMN-mRNA–LNP group, which may be attributable to the fact that SMN-mRNA–LNP induced higher levels of IgA in the sow colostrum than the inactivated PDCoV vaccine. However, the detailed mechanism underlying this phenomenon requires further study. After the sows delivered, the piglets in the SMN-mRNA–LNP group received higher levels of IgG, IgA, and neutralizing antibodies passively through breast milk than did the piglets in the inactivated PDCoV vaccine group, and after challenge, the piglets in the SMN-mRNA–LNP group achieved 100% (5/5) passive protection, whereas the piglets in the inactivated vaccine group achieved only 40% (2/5) passive protection. A pathological histological examination and immunohistochemical analysis showed that the intestinal tracts of the piglets in the SMN-mRNA–LNP group were almost devoid of PDCoV antigens and showed almost no pathological damage.

During a study of immunization against infectious gastroenteritis, Linda J. Saif showed that the secretory IgA (sIgA) component induced by intestinal immunization is essential for the prevention and control of TGEV infections in neonatal piglets, which led to the concept of the gut–mammary gland–sIgA axis (32). The essence of this axis is that the infection of the intestinal epithelium by pathogenic microorganisms results in the production of lymphoplasmacytic cells capable of synthesizing sIgA in the mesenteric lymph nodes, which can either return to the lamina propria of the intestinal tract or be transferred to the mammary gland. Lymphocytes in the basal layer of the mammary epithelium of immunized sows continuously secrete sIgA, which is not readily degraded by the enzymes in milk or the intestine. This confers long-lasting immune protection on suckling piglets. In the present study, both SMN-mRNA–LNP and S2P-mRNA–LNP induced higher titers of IgA antibodies than the inactivated vaccine, which may be related to the unique immune properties and immune mechanisms of mRNA vaccines. A related study found that exosomes containing S protein were present in humans after vaccination with BNT162b2 and persisted in plasma for several weeks after immunization (33). Moreover, the presence of antigens was consistently detected in the draining lymph nodes and serum after the vaccination of mice with BNT162b2 (34). Therefore, the very good immunoprotection conferred by the SMN-mRNA–LNP and S2P-mRNA–LNP vaccines in the present study may be attributable to the fact that, after immunization, the antigen is expressed in large quantities *in vivo* and reaches the intestinal mesenteric lymph nodes through the humoral circulation, which activates the gut–mammary gland–sIgA axis. This induces the production of high levels of IgA antibodies and their secretion into the colostrum. Therefore, if LNP can selectively deliver mRNA to the mesenteric lymph nodes, then mRNA–LNP may exert a better immunomodulatory effect. This will be the focus of future research.

In summary, the present study demonstrates that the single mRNA vaccine SMN-mRNA–LNP, which simultaneously expresses the three major structural proteins of PDCoV, provides better immunoprotection than the mRNA vaccine S2P-mRNA–LNP, which expresses only the individual S protein. It also efficiently avoids the complexity and high cost of the separate delivery of multiple proteins. We have also shown that PDCoV structural proteins N and M also play important roles in inducing the host’s immunoprotective response. Therefore, when developing coronavirus vaccines in the future, the N and M proteins should be considered in addition to the S protein, as important antigenic components of candidate vaccines whenever possible. The results of this study provide new ideas and a practical basis for the development of novel vaccines against porcine enteric coronaviruses in the future.

## MATERIALS AND METHODS

### Construction of plasmid DNA template

We used the sequence of the PDCoV virulent strain CH/XJYN/2016 as the reference and performed codon optimization of the gene sequences encoding the S (MN064712), M (OQ185377), and N proteins (OQ185376). We introduced two proline mutations (E855P and V856P) into the S protein to be expressed and designed and synthesized two plasmid templates for mRNA transcription *in vitro*: pS-2P and pSMN.

### mRNA production and LNP encapsulation

Using the linearized pS-2P and pSMN plasmids as templates, we performed *in vitro* transcription with the EasyCap Co-Transcriptional Capping Kit (Vazyme, Nanjing) to produce single-stranded mRNAs featuring the 5′-m7G Cap1 structure, poly(A) tail, and N1-methyl-pseudouridine modifications. The crude mRNA products were purified with a magnetic-bead-based method to remove the enzymes, DNA templates, and free nucleotides, yielding high-purity mRNA stocks. For LNP formulation, we prepared an ethanol solution containing a cationic lipid, 1,2-distearoyl-sn-glycero-3-phosphocholine (DSPC), cholesterol, and DMG–PEG 2000 in a molar ratio of 50:10:38.5:1.5. Using the NanoAssemblr Ignite system based on the NxGen microfluidic technology, the lipid mixture was combined with mRNA in 20 mM citrate buffer (pH 4.0) in a 1:3 volumetric flow rate ratio to achieve mRNA encapsulation. The mRNA–LNPs were then dialyzed against phosphate-buffered saline (PBS; pH 7.4) and then concentrated to the desired concentration with a 100-kD Amicon centrifugal ultrafiltration tube (Millipore, USA), yielding two final mRNA–LNP formulations, designated S2P-mRNA–LNP and SMN-mRNA–LNP.

### Western blotting analysis

293T cells at a cell density of about 80% were transfected with either S2P-mRNA–LNP or SMN-mRNA–LNP (2 µg/well), whereas the control wells were treated with Lipo3000 alone. All the cells were cultured in a 37°C incubator for 24 h. The cells were collected, and the cell lysates were subjected to polyacrylamide gel electrophoresis. The separated proteins were then transferred to a nitrocellulose membrane (Pall, USA) for western blotting analysis. They were probed with rabbit anti-S, anti-M, or anti-N polyclonal primary antibody (diluted 1:500) prepared in our laboratory, and then with horseradish peroxidase (HRP)-conjugated goat anti-rabbit immunoglobulin IgG secondary antibody (diluted 1:10,000; Zhongshan Golden Bridge Biotechnology, Beijing, China). The proteins were visualized with enhanced chemiluminescent substrate (ECL, Thermo Scientific, USA).

### Immunofluorescence assay

LLC-PK cells at a density of about 80% were transfected with S2P-mRNA–LNP or SMN-mRNA–LNP (2 µg/well), whereas the control wells were treated with Lipo3000 alone. All the cells were cultured in a 37°C incubator for 24 h. The medium was then discarded, and the cells were washed three times with PBS, fixed in 4% paraformaldehyde at 4°C for 1 h, permeabilized with 0.25% Triton X-100 at room temperature for 10 min, and blocked with 5% bovine serum albumin (BSA) for 1 h. They were then incubated with rabbit anti-S, -M, or -N polyclonal primary antibody, and then with Alexa-Fluor-488- or Alexa-Fluor-594-conjugated goat anti-rabbit IgG antibody as the secondary antibody. The cells were stained with 4′,6-diamidino-2-phenylindole (DAPI) for 5 min in the dark, washed three times with PBS, and observed with fluorescence microscopy.

### Mouse immunization assays

Eighteen 4- to 6-week-old specific-pathogen-free (SPF) female BALB/c mice were randomly allocated to three groups (6 mice/group) and immunized with S2P-mRNA–LNP, inactivated PDCoV vaccine, or PBS. The mice in the S2P-mRNA–LNP group were inoculated with a dose of 30 µg/mouse; those in the PDCoV-inactivated vaccine group were inoculated with a dose of 100 µL/mouse; and those in the control group were injected with PBS. The mice in each of the three groups were immunized once with a dorsal subcutaneous injection on days 0 and 14 (Fig. 2A), and blood was collected from each mouse before immunization and on days 14 and 28 after the initial immunization.

### Active immunization and protection assay in nursing piglets

Twenty 5-day-old nursing piglets were purchased from commercial pig farms with no history of PDCoV infection or vaccination. Indirect enzyme-linked immunoassays (ELISAs) and reverse transcription (RT)–real-time quantitative PCR (qPCR) were performed on serum and fecal swabs from both the sows and piglets to ensure PDCoV negativity. The 20 5-day-old nursing piglets were randomly divided into four groups (five piglets/group), and immunized with 2P-mRNA–LNP, SMN-mRNA–LNP, inactivated PDCoV vaccine, or PBS. The S2P-mRNA–LNP and SMN-mRNA–LNP group piglets were inoculated with a dose of 40 µg/piglet, the inactivated PDCoV vaccine group piglets with a dose of 1 mL/piglet (viral load before inactivation: 1.0 × 10^6^.^5^ 50% median tissue culture infective doses [TCID_50_]/mL), and the control group with PBS. The piglets in each group were immunized once on day 0 and once on day 14 (Fig. 3A), and serum was collected before immunization and on days 14 and 28 after the initial immunization. On day 28 after immunization, all the piglets were inoculated orally with 8 mL ×10^4.0^ TCID_50_/mL CH/XJYN/2016 (35). After inoculation with PDCoV, the piglets were observed for clinical symptoms and tested for the excretion of viral RNA with fecal swabs on seven consecutive days. Clinical signs of diarrhea were scored as normal = 0; pasty = 1; semiliquid = 2; and liquid = 3.

### Passive immunization and protection assay in nursing piglets

Nine sows confirmed to be double-negative for both PDCoV antigens and antibodies were randomly divided into three groups (three sows/group). At 42 days before farrowing, the sows in the three groups were injected with SMN-mRNA–LNP at a dose of 100 µg/sow, inactivated PDCoV vaccine (viral load of 1.0 × 10^6^.^5^ TCID_50_/mL before inactivation) at a dose of 2 mL/sow, or PBS. All sows received a booster immunization 21 days after the primary immunization. Serum samples were collected before immunization, on day 21 after the primary immunization, and on the day of farrowing. Colostrum was collected after farrowing for antibody detection. Five days after farrowing, five piglets were randomly selected from each group of sows (ensuring that piglets from different sows within the same group were chosen to minimize the influence of any variations in individual sows). Each piglet was orally challenged with 1 mL of 10^4.0^ TCID_50_/mL CH/XJYN/2016. After the viral challenge, the piglets were monitored daily for clinical symptoms and to assess viral shedding levels and diarrhea scores across the experimental groups.

### Specific antibody detection

An indirect ELISA was used to determine the levels of specific antibodies directed against the S, M, or N protein in the sera and colostrum (26). Briefly, purified S, M, or N proteins were used to coat separate 96-well enzyme-labeled plates (Corning, USA), which were blocked with 5% skimmed milk powder at 37°C for 2 h. Then, a 100 µL doubly serially diluted serum or colostrum sample was added to each well and incubated at 37°C for 1 h. The liquid in the wells was discarded, and they were washed three times with PBS containing Tween 20 (PBST), and 100 µL HRP-conjugated goat anti-pig IgA or IgG secondary antibody was added to each well and incubated at 37°C for 1 h. The liquid in the wells was discarded, and the wells were washed three times with PBST. TMB substrate solution (100 µL) (Solarbio, Beijing, China) was added to each well and allowed to react for 10 min at 37°C in the dark. The reaction was terminated by the addition of 100 µL of 2 M H_2_SO_4_ to each well, and the absorbance of each well was measured at 450 nm.

### Neutralization assay

The serum neutralization test was performed with the fixed virus-diluted serum method, as follows. A 96-well plate containing 70%–80% LLC-PK cells was prepared in advance. The serum was inactivated in a water bath at 56°C for 30 min and then made twofold serial doubling dilutions in a 96-well microtiter plate, with four replicate wells for each dilution. Diluted virus solution (200 TCID_50_) was added to each well and mixed well, and the plate was placed in a 37°C 5% CO_2_ incubator for 1 h. A virus-positive control and a normal-cell control were prepared. The virus-positive control was set up with four different concentrations of 200 TCID_50_ (all diseased), 20 TCID_50_, 2 TCID_50_, or 0.2 TCID_50_ (not diseased). The cells were washed three times with PBS, and 100 µL of the virus–serum mixture was added to each well. The plate was incubated at 37°C in a 5% CO_2_ incubator for 1 h. After incubation, the liquid in the wells was discarded, the cells were washed three times with PBS, and 200 µg of maintenance medium (containing 20 µg/mL trypsin) was added to each well. Any cytopathic effect (CPE) was observed and recorded daily. Neutralizing antibody titers were calculated as the reciprocal of the highest serum dilution that inhibited 50% CPEs.

### Flow-cytometric analysis

On day 28 after immunization, the spleens of the mice were collected, and lymphocyte suspensions were prepared with an animal lymphocyte isolation kit (Solarbio). The lymphocyte suspension was diluted to 5 × 10^6^ cells/mL with RPMI 1640 medium (Gibco, USA). The appropriate amounts of fluorescein isothiocyanate (FITC)-conjugated anti-mouse CD3 antibody (BioLegend, cat.: 100204), phycoerythrin (PE)-conjugated anti-mouse CD4 antibody (BioLegend, cat.: 100408), or allophycocyanin (APC)-conjugated anti-mouse CD8a antibody (BioLegend, cat.: 100713) was added to 100 µL of lymphocyte suspension and mixed well. The cells were incubated at 4°C for 30 min. They were then washed three times with ice-cold PBS to remove any unbound antibodies. A cell phenotype analysis was performed with a CytoFLEX LX 5L19C flow cytometer (Beckman Coulter Inc.).

### Lymphocyte proliferation assay

We added the prepared lymphocyte suspension to 96-well cell culture plates at 100 µL/well, and added S protein at a final concentration of 5 µg/mL to stimulate lymphocyte proliferation, with six replicate wells per sample. The stimulated wells were used as the positive control, and wells without stimulant were cell control wells. We placed the 96-well plates at 37°C in a 5% CO_2_ cell culture incubator for 72 h. The cell culture supernatants were collected for a cytokine detection assay, and the medium was replaced with fresh medium. Cell Counting Kit-8 (CCK-8) solution (10 µL) (Beyotime, China) was added to each well, and the cells were incubated at 37°C for 2 h with shaking for 1 min. The optical density (OD) of each well was measured at 490 nm with a spectrophotometer, and the stimulation index (SI) was calculated with the formula SI = (OD_490_ of the stimulated wells − OD_490_ of the blank control wells)/(OD_490_ of the control wells − OD_490_ of the blank control wells).

### Detection of cytokine expression levels

After the lymphocytes were stimulated with S protein for 72 h, the lymphocyte culture supernatant was collected. Cytokine ELISA kits were used to measure the levels of interferon gamma (IFN-γ), interleukin 2 (IL-2), IL-4, IL-21, and transforming growth factor beta 1 (TGF-β1) in the supernatants, according to the manufacturers’ instructions.

### Pathohistological and immunohistochemical assays

The ileums of the piglets were collected and fixed in 10% neutral formaldehyde. The fixed tissues were dehydrated in a JT-12S automatic tissue dehydrator, embedded, and sectioned. The sections were dewaxed, rehydrated, stained with hematoxylin for 10-20 min, hydrochloric acid alcohol differentiated for 5–10 s, washed with water for 1-3 min, and then placed in warm water (50°C) or weak alkaline aqueous solution until a blue color appeared. The sections were treated with 85% alcohol for 3–5 min, stained with eosin for 3–5 min, washed with water for 3–5 s, dehydrated through a gradient of alcohol, rendered transparent with xylene, sealed with neutral resin, and then microscopically examined. A Panoramic 250 Flash III DX Digital Scanner was used to capture images of the sections for the observation of specific lesions. A 1:100 dilution of a monoclonal antibody directed against the PDCoV N protein prepared in our laboratory was used as the primary antibody, HRP-conjugated goat anti-mouse IgG was used as the secondary antibody, and 3,3′-diaminobenzidine (DAB), applied for 2 min, was used for the color reaction. Images of the sections were acquired with the BA200 Digital Digital Trinocular Camera Microscopic Video Camera System.

### Statistical analysis

For all statistical analyses, we used the GraphPad Prism software. The differences between groups were analyzed by mixed-effect analysis or one-way analysis of variance (ANOVA) and Tukey’s multiple comparison test for statistical significance. *P* > 0.05 not statistically significantly different; *P < 0.05 significantly different; **P < 0.01, highly significantly different; ***P < 0.001, very highly significantly different.

## Data Availability

All data generated or analyzed during this study are included in this article and its supplemental material. The data supporting the results of this study are available from the corresponding author upon reasonable request.

## SUPPLEMENTAL MATERIAL

Table S1 jvi.00849-25-s0001.docx Clinical symptom scores of piglets.

ASM does not own the copyrights to Supplemental Material that may be linked to, or accessed through, an article. The authors have granted ASM a non-exclusive, world-wide license to publish the Supplemental Material files. Please contact the corresponding author directly for reuse.
