# Supplementary material for: Single self-cleaving mRNA vaccine expressing multiple viral structural proteins elicits robust immune responses and protects nursing piglets against PDCoV infection
Source: J Virol. 2025 Aug 6;99(9):e00849-25. doi: 10.1128/jvi.00849-25 (PMC12455934; doi:10.1128/jvi.00849-25)
Supplement: Table S1 — Clinical symptom scores of piglets. [file jvi.00849-25-s0001.docx]

**Table S1 Summary of the clinical symptoms scores of piglets challenged with 1 ml × 10^4.0^ TCID_50_/ml PDCoV CH/XJYN/2016 after passive immunization**

| dpi | SMN-mRNA-LNP (n=5) | | | |  | PDCoV inactivated (n=5) | | | |  | PBS (n=5) | | | |
| --- | --- | --- | --- | --- | --- | --- | --- | --- | --- | --- | --- | --- | --- | --- |
|  | Vomiting | Diarrhea | Inappetence | Apatheia |  | Vomiting | Diarrhea | Inappetence | Apatheia |  | Vomiting | Diarrhea | Inappetence | Apatheia |
| 0 | － | － | － | － |  | － | － | － | － |  | － | － | － | － |
| 1 | － | － | － | － |  | － | － | － | － |  | － | － | － | － |
| 2 | － | － | － | － |  | ＋ | ＋ | ＋ | ＋ |  | ＋ | ＋＋ | ＋ | ＋ |
| 3 | － | － | － | － |  | ＋ | ＋＋ | ＋ | ＋ |  | ＋ | ＋＋＋ | ＋＋ | ＋＋ |
| 4 | － | － | － | － |  | ＋ | ＋＋ | ＋＋ | ＋＋ |  | ＋＋ | ＋＋＋ | ＋＋＋ | ＋＋ |
| 5 | － | － | － | － |  | ＋ | ＋＋ | ＋ | ＋＋ |  | ＋＋ | ＋＋＋ | ＋＋＋ | ＋＋ |
| 6 | － | － | － | － |  | ＋ | ＋ | ＋ | ＋ |  | ＋ | ＋＋ | ＋＋ | ＋＋ |
| 7 | － | － | － | － |  | ＋ | ＋ | ＋ | ＋ |  | ＋ | ＋ | ＋ | ＋＋ |
| 8 | － | － | － | － |  | － | － | － | － |  | － | ＋ | ＋ | ＋ |
| 9 | － | － | － | － |  | － | － | － | － |  | － | － | － | － |
| 10 | － | － | － | － |  | － | － | － | － |  | － | － | － | － |

“－” indicates that the piglets have no clinical symptoms of vomiting, diarrhea, inappetence, and apatheia.

“＋” indicates that the piglets have mild clinical symptoms of vomiting, diarrhea, inappetence, and apatheia.

“＋＋” indicates that the piglets have moderate clinical symptoms of vomiting, diarrhea, inappetence, and apatheia.

“＋＋＋” indicates that the piglets have severe clinical symptoms of vomiting, diarrhea, inappetence, and apatheia.
